# E-cadherin-deficient cells have synthetic lethal vulnerabilities in plasma membrane organisation, dynamics and function

**DOI:** 10.1007/s10120-018-0859-1

**Published:** 2018-07-31

**Authors:** Tanis D. Godwin, S. Thomas Kelly, Tom P. Brew, Nicola M. Bougen-Zhukov, Andrew B. Single, Augustine Chen, Cassie E. Stylianou, Lawrence D. Harris, Sophie K. Currie, Bryony J. Telford, Henry G. Beetham, Gary B. Evans, Michael A. Black, Parry J. Guilford

**Affiliations:** 1 Cancer Genetics Laboratory, Centre for Translational Cancer Research (Te Aho Matatū), Department of Biochemistry, University of Otago, Dunedin, New Zealand; 2 The Ferrier Research Institute, Victoria University of Wellington, Wellington, New Zealand; 3 Parry Guilford Cancer Genetics Laboratory, Department of Biochemistry, University of Otago, PO Box 56, Dunedin, 9016 New Zealand

**Keywords:** Diffuse gastric cancer, Synthetic lethality, E-cadherin, Plasma membrane, *CDH1*

## Abstract

**Background:**

The E-cadherin gene (*CDH1*) is frequently mutated in diffuse gastric cancer and lobular breast cancer, and germline mutations predispose to the cancer syndrome Hereditary Diffuse Gastric Cancer. We are taking a synthetic lethal approach to identify druggable vulnerabilities in *CDH1*-mutant cancers.

**Methods:**

Density distributions of cell viability data from a genome-wide RNAi screen of isogenic MCF10A and MCF10A-*CDH1*^−/−^ cells were used to identify protein classes affected by *CDH1* mutation. The synthetic lethal relationship between selected protein classes and E-cadherin was characterised by drug sensitivity assays in both the isogenic breast MCF10A cells and *CDH1*-isogenic gastric NCI-N87. Endocytosis efficiency was quantified using cholera toxin B uptake. Pathway metagene expression of 415 TCGA gastric tumours was statistically correlated with *CDH1* expression.

**Results:**

MCF10A-*CDH1*^−/−^ cells showed significantly altered sensitivity to RNAi inhibition of groups of genes including the PI3K/AKT pathway, GPCRs, ion channels, proteosomal subunit proteins and ubiquitinylation enzymes. Both MCF10A-*CDH1*^−/−^ and NCI-N87-*CDH1*^−/−^ cells were more sensitive than wild-type cells to compounds that disrupted plasma membrane composition and trafficking, but showed contrasting sensitivities to inhibitors of actin polymerisation and the chloride channel inhibitor NS3728. The MCF10A-*CDH1*^−/−^ cell lines showed reduced capacity to endocytose cholera toxin B. Pathway metagene analysis identified 20 Reactome pathways that were potentially synthetic lethal in tumours. Genes involved in GPCR signalling, vesicle transport and the metabolism of PI3K and membrane lipids were strongly represented amongst the candidate synthetic lethal genes.

**Conclusions:**

E-cadherin loss leads to disturbances in receptor signalling and plasma membrane trafficking and organisation, creating druggable vulnerabilities.

**Electronic supplementary material:**

The online version of this article (10.1007/s10120-018-0859-1) contains supplementary material, which is available to authorised users.

## Introduction

*CDH1* is a tumour suppressor gene that encodes E-cadherin, a homophilic cell-to-cell adhesion protein that is localised to the adherens junction on the basolateral surface of epithelial cells. In addition to cell adhesion, E-cadherin plays central roles in differentiation, cell polarity, migration, the detection of cell–cell tension and cell survival signalling [1]. Somatic mutations in *CDH1* occur frequently in diffuse gastric cancer (DGC) and lobular breast cancer (LBC) and its downregulation is a hallmark of the epithelial–mesenchymal transition [2, 3]. In addition, germline *CDH1* mutations genetically characterise the inherited cancer syndrome Hereditary Diffuse Gastric Cancer (HDGC) [4]. Carriers of pathogenic *CDH1* mutations from HDGC families have a ~ 70% lifetime risk of developing advanced DGC and female carriers have an additional ~ 40% risk of LBC [5, 6]. To develop novel drugs for the chemoprevention and treatment of E-cadherin-deficient tumours, we are taking a synthetic lethal (SL) approach to identify vulnerabilities in these cancers. We have previously conducted a genome-wide siRNA screen and a 4000 compound ‘known drug’ screen in isogenic breast MCF10A cells with and without E-cadherin expression to identify vulnerabilities in the MCF10A-*CDH1*^−/−^ cells that could be exploited in an SL manner [7]. MCF10A is a non-malignant breast cell line with relatively few cytogenetic abnormalities and mutations [8, 9]. It is therefore a suitable reference line for studies that explore normal protein interactions and also an informative model for HDGC chemoprevention studies, particularly since equivalent non-malignant stomach cell lines are not available. Compared to MCF10A cells, MCF10A*-CDH1*^−/−^ cells have modest changes in cell morphology but show striking differences in the organisation of both the actin and microtubule cytoskeletons [10]. Our genome-wide siRNA screen on this isogenic line identified large numbers of potential SL interactions with E-cadherin, particularly in GPCR signalling and cytoskeletal functions. MCF10A*-CDH1*^−/−^ cells were also more sensitive to multiple drugs including antagonists of PI3K, c-SRC and histone deacetylases. However, a unifying SL mechanism was not established. To elucidate the mechanisms underpinning E-cadherin’s diverse SL interactions and the subsequent vulnerabilities in *CDH1*-null cancers, we have now extended our analysis of isogenic cell line pairs and applied a novel bioinformatic approach that statistically queries tumour genome-wide expression data for potential SL pathways. Our data supports a model in which rearrangement of the actin cytoskeleton following abrogation of the adherens junction [10] leads to disruption of normal plasma membrane organisation and dynamics. We provide evidence which suggests that this disruption leads to wide-ranging disturbances in receptor signalling and plasma membrane trafficking, which in turn create druggable synthetic lethal vulnerabilities in E-cadherin-null cells.

## Materials and methods

### Cell viability ratios

Data on cell viability after gene knockdown were previously generated via a high-throughput siRNA screen in the breast MCF10A and MCF10A*-CDH1*^−/−^ isogenic lines [7]. For each gene, a *viability ratio* (MCF10A/MCF10A*-CDH1*^−/−^) was calculated to indicate the relative impact of gene knockdown on cell viability in the two cell lines.

### Visualisation of viability ratios

The statistical software R was used to generate density plots summarising the distribution of viability ratios for specific lists of genes [11]. Lists were obtained from a variety of sources and are noted in the figure legends. Extracellular ligands were not included in the signalling pathway lists because of the artificial cell culture environment. To identify statistical differences between the viability ratio distributions for the selected gene sets and the distribution for all 18,120 genes included in the siRNA screen, the Kolmogorov-Smirnov test was used.

### Cancer genome atlas gene expression data

RNA-seq data from the Cancer Genome Atlas (TCGA) Stomach Adenocarcinoma (STAD) project was downloaded from the International Cancer Genome Consortium data portal (version 25, September 2017). Per-gene read count data were processed in R [11], using the RNA-seq workflow described in the limma package users guide. Normal tissue samples were removed from the analysis, leaving expression data for 20,502 genes across 415 gastric tumours.

### Metagene creation

The reactome.db R package was used to obtain information about gene membership of Reactome pathways [12]. For each pathway comprising at least five genes, singular value decomposition (SVD) was used to generate a metagene (with per-gene expression data standardised to have mean 0 and standard deviation 1 prior to performing SVD) using the first eigenvector for that pathway.

### Computational identification of SL relationships

To identify pathway metagenes that exhibited a potential synthetic lethal association with *CDH1*, samples were binned into tertile groups (bottom 1/3, middle 1/3, top 1/3) for each quantity of interest (metagene value or *CDH1* expression), and a chi-squared statistic was calculated as a measure of association between metagene tertiles and *CDH1* tertiles. To account for inter-gene correlation that was not pathway-specific, resampling was used to generate a null distribution of the chi-squared statistics. For each pathway size, k (i.e., number of genes in the pathway), a random sample of k genes was taken, and their expression values were used to generate a metagene as described above. A chi-squared statistic was then generated for the resampled data, and this process was repeated 500,000 times per pathway to generate a null distribution for each pathway metagene. Empirical *p* values were then calculated for each pathway metagene (by counting the number of times that the resampled chi-squared statistics exceeded the observed value), and these *p* values were then adjusted for multiple testing using the Benjamini and Hochberg False Discovery Rate (FDR) controlling procedure. Pathway metagenes with a FDR-adjusted *p* value below 0.2 were considered to exhibit a significant association with *CDH1* levels. Hierarchical clustering was carried out using the heatmap.2 function inside the ggplot2 package.

### Drug titrations

Latrunculin B, cytochalasin D, methyl-β-cyclodextrin and amphotericin B were obtained from Sigma-Aldrich and bafilomycin A1, atorvastatin, AZD5363, otenabant and PI103 from SelleckChem. NS3728 was synthesised at the Ferrier Institute. The NCI-N87 gastric cancer cell line was obtained from ATCC. NCI-N87-*CDH1*^−/−^ was generated by homozygous frameshift deletion of base c.1553 in exon 10 using CRISPR-Cas9 (A Chen, manuscript in preparation). Drug treatments were largely carried out as previously described [7]. Briefly, MCF10A and MCF10A*-CDH1*^−/−^ cells were seeded in black-walled, clear-bottom 96 well plates (Corning, Corning, NY) at a density of 4000 cells/well in a volume of 100 µl. NCI-N87 and NCI-N87-*CDH1*^−/−^ (passage ≤ 10) were seeded at 10,000 cells/ml. After a 24 h incubation, 10 µl drug (dissolved in vehicle to a 20 mM stock and diluted in complete media) was added to each well. After a further 48 h, cells were fixed and stained with 1 µg/mL Hoechst 33,342, 0.25% paraformaldehyde and 0.075% saponin in PBS [13]. Six fields/well at 4× magnification were captured using the Cytation 5 imager (Biotek). Nuclei were counted using Gen5 (Biotek) and normalised to the vehicle control for each cell line.

### Cholera toxin endocytosis assay

MCF10A and MCF10A-*CDH1*^−/−^ cells were seeded at 4000 cells per well in 96-well, black-walled, clear-bottom tissue culture plates in 100 µL cholera toxin-deficient complete growth medium. Plates were incubated at RT for 30 min then at 37 °C (5% CO_2_) for a further 48 h. The medium was then replaced with 50 µL cholera toxin-deficient growth medium supplemented with 10 µg/mL Alexa Fluor 488-Cholera Toxin Subunit B (Thermo Fisher Scientific, Waltham, MA) and plates incubated for 30 min at 37 °C (5% CO_2_). Cells were washed twice with PBS and fixed with 0.25% paraformaldehyde in PBS with 1 µg/mL Hoechst 33,342 (Thermo Fisher Scientific) overnight at RT in the dark. Fluorescent microscopy was performed at 10× magnification using a Nikon Eclipse Ti inverted microscope, and at least 15 images per well were acquired with a DS-QiMc camera. Hoechst-stained nuclei were quantified for a total cell count using CellProfiler™ (Broad Institute). ImageJ was used to measure the fluorescent intensity of cholera toxin and subtract background fluorescence [14].

## Results

### Different protein classes show contrasting SL distributions

**Fig. 1:**
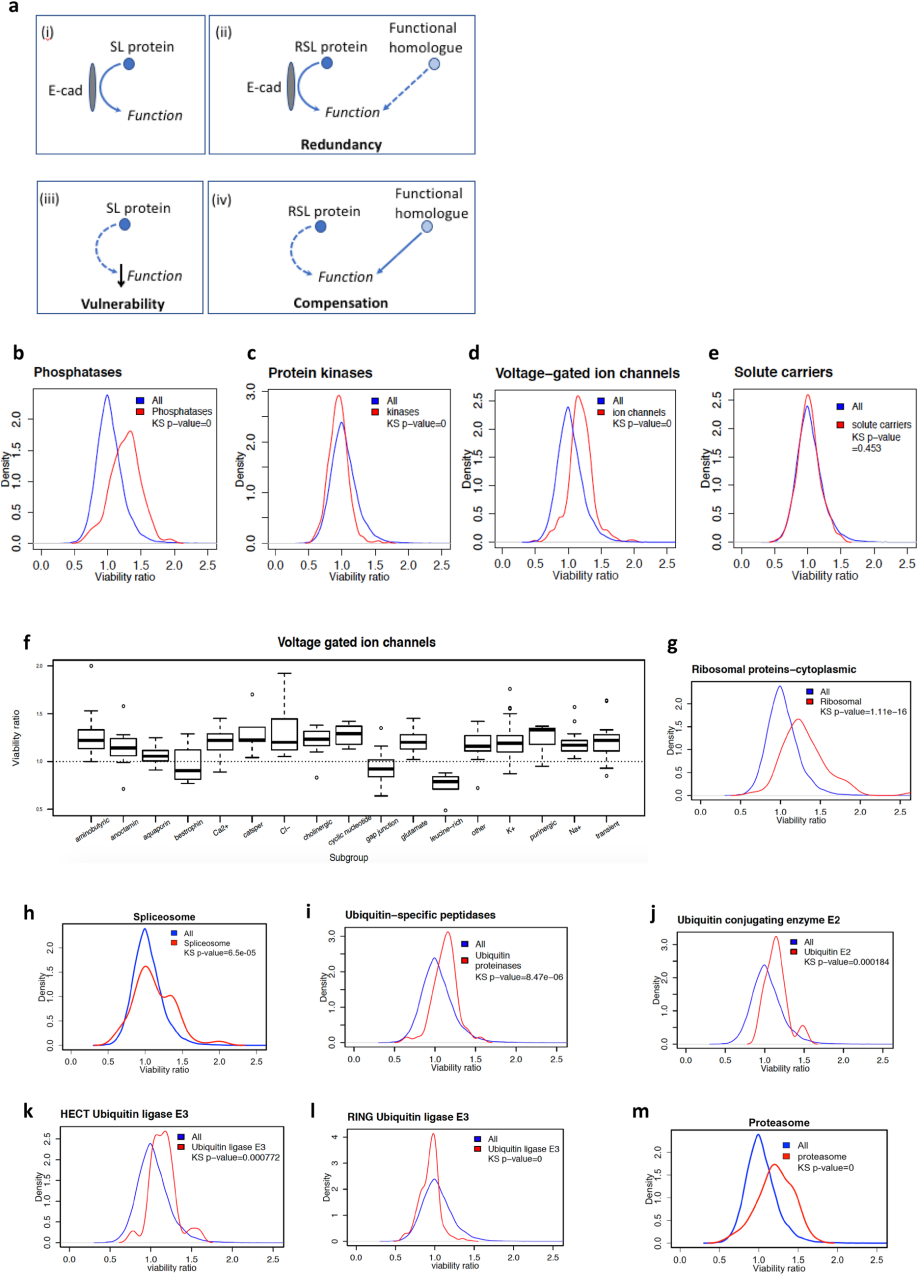
**a** Model of SL and RSL interactions. SL and RSL proteins both have functions affected by E-cadherin activity; however, RSL proteins have the potential to be compensated for by functional homologues (i–ii). When E-cadherin is lost, inhibition of an SL protein leads to reduced cell fitness (iii), whereas the inhibition of an RSL protein has less effect because its function is compensated for by the increased activity of the functional homologue (iv). **b**–**e** Density distributions of MCF10A-*CDH1*^−/−^/MCF10A viability ratios following siRNA knockdown of families of phosphatases, protein kinases, voltage-gated ion channels and solute carriers. Lists were obtained from a variety of sources: phosphatases from the human dephosphorylation database (http://www.Koehn.embl.de/depod), protein kinases from UniProt (http://www.uniprot.org) and voltage-gated ion channels and solute carriers from the Human Gene Nomenclature Committee (http://www.genenames.org). Distributions were generated in R using knockdown data from the MCF10A and MCF10A-*CDH1*^−/−^ cell line pair. The distribution of all 18,120 genes in the siRNA screen is represented by the blue lines (‘All’). The statistical significance of any differences between the distributions of all data (blue) and the functional group (red) was determined using the Kolmogorov-Smirnov test. KS *p* value = 0 equates to a *p* value of < 2.2 × 10^−16^. **f** Boxplot expanding the viability ratios of voltage-gated ion channels into functional subgroups. With the exception of the bestrophin, gap junction and leucine-rich subgroups, all groups were RSL, with a median viability ratio > 1. **g**–**m** Density distributions of cytoplasmic ribosomal proteins, spliceosome proteins, ubiquitin-specific peptidases, E2 ubiquitin-conjugating enzymes, HECT family ubiquitin E3 ligases, RING family ubiquitin E3 ligases and proteasome subunits. Lists were obtained from sources including ribosomal proteins—the ribosomal protein database (ribosome.med.miyazaki-u.ac.jp), ubiquitin-associated peptidases and E2 ubiquitin-conjugating enzymes—the Human Gene Nomenclature Committee (http://www.genenames.org), and E3 ubiquitin ligases from the National Heart Lung and Blood Institute (https://hpcwebapps.cit.nih.gov/ESBL/Database/E3-ligases/)

**Fig. 2:**
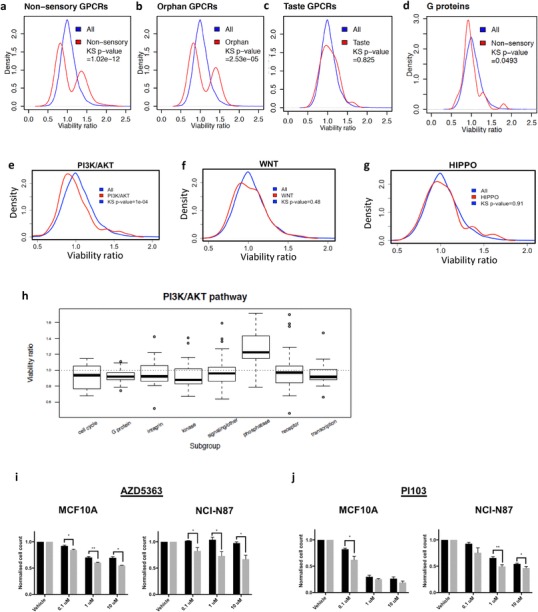
**a–d** Density distributions of the *CDH1*^−/−^/MCF10A viability ratios for non-sensory GPCRs, orphan GPCRs, taste GPCRs and G proteins. **e–g** Density distributions of the *CDH1*^−/−^/MCF10A viability ratios for signalling pathways associated with intact adherens junctions: PI3K/AKT, WNT pathway and HIPPO pathway. Pathway genes were obtained from KEGG. The statistical significance was determined using the Kolmogorov–Smirnov test. **h** Boxplot of the functional subgroups in the PI3K/AKT pathway. Note that all subgroups with the exception of the phosphatases are shifted in the synthetic lethal direction (viability ratio < 1). **i–j** Normalised cell counts 48 h after treatment with serial dilutions of AZD5363 and PI103. For each drug, both the MCF10A and NCI-N87 isogenic pair are shown (wild-type black bars; *CDH1*^−/−^ grey bars). Six fields/well at 4× magnification were captured using the Cytation 5 imager (Biotek). Nuclei were counted using Gen5 (Biotek) and normalised to the vehicle control for each cell line

We previously carried out a genome-wide siRNA screen in isogenic breast MCF10A cells with and without E-cadherin expression and observed that GPCRs and cytoskeletal proteins frequently displayed an SL relationship with E-cadherin [7]. To elucidate the mechanisms underpinning synthetic lethality in E-cadherin-deficient cells, we have now extended our analysis of the siRNA screen data to include genes with the reverse effect to synthetic lethality, that is, genes whose siRNAs reduced the viability of MCF10A cells more than MCF10A*-CDH1*^−/−^ cells. We have termed these genes ‘reverse synthetic lethal’ (RSL). We hypothesise that RSL proteins have direct or indirect interactions with E-cadherin, but *CDH1*^−/−^ cells have compensated for the loss of this interaction, making them less sensitive to knockdown of RSL proteins (relative to wild-type cells). This adaption is most likely to have occurred through the activation of one or more proteins with overlapping function (i.e., functional redundancy). In contrast to these RSL proteins, we hypothesise that MCF10A*-CDH1*^−/−^ cells have been unable to fully compensate for the loss of the interaction between E-cadherin and any given SL protein, exposing a vulnerability (summarised in Fig. 1a). Although RSL proteins may not represent useful drug targets on their own, compounds which also inhibit the functional homologue may prove to be SL. Regardless, the identity of RSL proteins provides a fuller understanding of the impact of E-cadherin loss on epithelial cells. 1648 genes met our RSL threshold of a MCF10A*-CDH1*^−/−^/MCF10A viability ratio of ≥ 1.3. Using the DAVID Functional Annotation Clustering tool v6.8 [15], the most enriched functional cluster identified amongst RSL genes was a group of terms associated with phosphatases (enrichment score = 14.88; Table 1). Of 239 phosphatases in the Human Dephosphorylation Database, 224 were included in our siRNA screen. A density distribution of the MCF10A*-CDH1*^−/−^/MCF10A viability ratios for these 224 phosphatases produced a single peak with the highest density occurring at a viability ratio of 1.34 (Fig. 1b). This distribution was shifted significantly (*p* < 2.2 × 10^−16^) in the RSL direction compared to the distribution of all 18,120 genes in the siRNA screen which displayed a single peak centered around a MCF10A*-CDH1*^−/−^/MCF10A ratio of 1.00. In contrast, the overall density distribution of 496 protein kinases in the siRNA screen showed a small but highly significant shift (*p* < 2.2 × 10^−16^) towards synthetic lethality with the peak of the density distribution at 0.95 (Fig. 1c). The contrasting cell viability distribution of kinase and phosphatase siRNAs is likely to be related to the greater functional promiscuity of phosphatases [16]. Ion channels were also highly enriched in the gene set analysis of RSL genes (enrichment score = 10.93; Table 1). Consistent with this observation, the MCF10A*-CDH1*^−/−^/MCF10A density distribution of 161 voltage-gated ion channels in the siRNA screen was significantly right-shifted from the overall distribution of all 18,120 genes with a peak at 1.14 (*p* < 2.2 × 10^−16^; Fig. 1d). In contrast, the distribution of 370 solute carrier genes was not significantly different to the distribution of all 18,120 genes (*p* = 0.45; Fig. 1e). The RSL phenotype of the ion channels was observed in most voltage-gated ion channel subgroups with the exception of small families of gap junction (*n* = 20), bestrophin (*n* = 4) and leucine-rich repeat channel (*n* = 5) proteins which showed a contrasting trend towards a SL phenotype (Fig. 1f). Terms associated with ribosomes, splicing and proteasomes were also enriched in the RSL gene set analysis (Table 1). Cytoplasmic ribosomal proteins (*n* = 78) and spliceosome proteins (*n* = 132) showed a corresponding significant RSL shift in the MCF10A*-CDH1*^−/−^/MCF10A density distributions (Fig. 1g, h), as did ubiquitin-specific peptidases (*n* = 55), E2 ubiquitin conjugating enzymes (*n* = 36), HECT E3 ubiquitin ligases (*n* = 25) and proteasome complex proteins (*n* = 43) (Fig. 1i, j, k, m). In contrast, the RING E3 ubiquitin ligases (*n* = 299) were not RSL, but instead featured a large SL shoulder in the MCF10A*-CDH1*^−/−^/MCF10A ratios centered at approximately 0.8 (*p* < 2.2 × 10^−16^) (Fig. 1l). Surprisingly, GPCR signalling terms were highly enriched amongst the RSL gene functions (enrichment score = 14.33; Table 1) just as they were in the SL gene functions [7]. Of the 1409 GPCRs identified by the Human Gene Nomenclature Committee, 721 were represented in the siRNA screen, including 245 non-sensory, 86 orphan, 363 olfactory GPCRs and 27 taste GPCRs. Consistent with the gene ontology analyses, the MCF10A*-CDH1*^−/−^/MCF10A density distributions for the 245 non-sensory GPCRs had a bimodal distribution, with peaks at viability ratios of 0.82 and 1.37 (*p* = 1.0 × 10^−12^; Fig. 2a). The left peak contained the SL GPCRs identified previously and the right peak comprised the set of RSL GPCRs. A similar bimodal distribution was also observed for the 86 orphan GPCRs (peaks at 0.82 and 1.39; Fig. 2b). In contrast, the viability ratio distribution of the taste and olfactory GPCRs (which have negligible expression in breast cells) predictably presented as single peaks centered at viability ratios of 0.97 (Fig. 2c) and 1.07, respectively. The density distributions for a set of 35 guanine nucleotide binding protein (G-protein) genes showed a single major peak that was shifted in the SL direction (max = 0.92, *p* = 0.05; Fig. 2d). Together, the above results demonstrate that whole classes of membrane receptors, channels and protein-modifying enzymes are influenced by E-cadherin loss.

**Table 1:** Pathway enrichment analysis of reverse synthetic lethal gene families

Enrichment score	UniProt keywords or sequence features	Adj. *p* value
14.88	Protein phosphatase	4.2E−38
14.33	G-protein coupled receptor	1.2E−17
	Receptor	2.5E−17
10.93	Ion channel	3E−18
	Ion transport	1.4E−10
10.55	Receptor	2.5E−17
	Glycoprotein	1E−11
9.34	Ribonucleoprotein	3E−11
	Ribosomal protein	9.4E−10
9.04	Glycoprotein	1E−11
	Disulfide bond	7.3E−11
5.2	Spliceosome	1.3E−07
	mRNA splicing	3.5E−06
4.63	Metal ion-binding site: manganese 1; via carbonyl	6.3E−03
	Manganese	2.2E−01
4.46	Protease	3.5E−06
	Zymogen	1.5E−05
4.06	Metal ion-binding site: iron	8.3E−01
	Metal ion-binding site: manganese	9.8E−01
4.01	Palmitate	1.8E−03
	Lipoprotein	4.7E−02
4.00	Threonine protease	1.0E−06
	Proteasome	1.1E−04

The most significant UniProt Keywords for each enrichment cluster from a DAVID ontology analysis of genes with a *CDH1*^−/−^/MCF10A viability ratio ≥ 1.3 are shown. Where no UniProt Keyword was listed, the UniProt Sequence Feature was used

### The PI3K/AKT pathway is an important mediator of E-cadherin synthetic lethality

To determine whether we could also detect SL effects in major signalling pathways, we first examined the MCF10A*-CDH1*^−/−^/MCF10A viability ratio density distributions for three cell survival pathways that are associated with E-cadherin-mediated cell-to-cell adhesion: PI3K/AKT, WNT and HIPPO [17–22]. Using KEGG pathway genes, the PI3K/AKT pathway (*n* = 224) showed a significant SL shift (peak = 0.90, *p* = 1.3 × 10^− 4^; Fig. 2e) while no significant shift in distribution was observed for either the WNT or HIPPO pathways (*p* = 0.48 and 0.91, respectively; Fig. 2f, g). Separation of the PI3K/AKT pathway genes into functional subgroups showed that the SL effect occurred across most functional groups in the pathway including plasma membrane receptors, integrins and G proteins (Fig. 2h). The only exception to these SL effects was phosphatases which were strongly RSL. The NFKB and MAPK pathways, which are indirectly associated with adherens junction signalling, showed distributions that were not significantly different to the overall 18,120 gene distribution (*p* = 0.18 and 0.86, respectively; results not shown). Of the other major KEGG cancer pathways, MTOR, RAS, TNF, JAK-STAT and focal adhesion signalling pathways also showed significant SL distributions, but this synthetic lethality could be largely attributed to genes which overlapped with the canonical PI3K/AKT pathway (Suppl. Fig. 1). The observed synthetic lethality was further validated by testing the PI3K antagonist PI103 and the pan-AKT antagonist NS3728 on both the MCF10A isogenic cell line pair and a recently derived *CDH1* isogenic gastric NCI-N87 pair (A. Chen, manuscript in preparation). Both drugs caused a significant SL effect in the two cell line pairs (Fig. 2i, j), consistent with the siRNA data.

### *CDH1*^−/−^ cells show changes in membrane trafficking

**Fig. 3:**
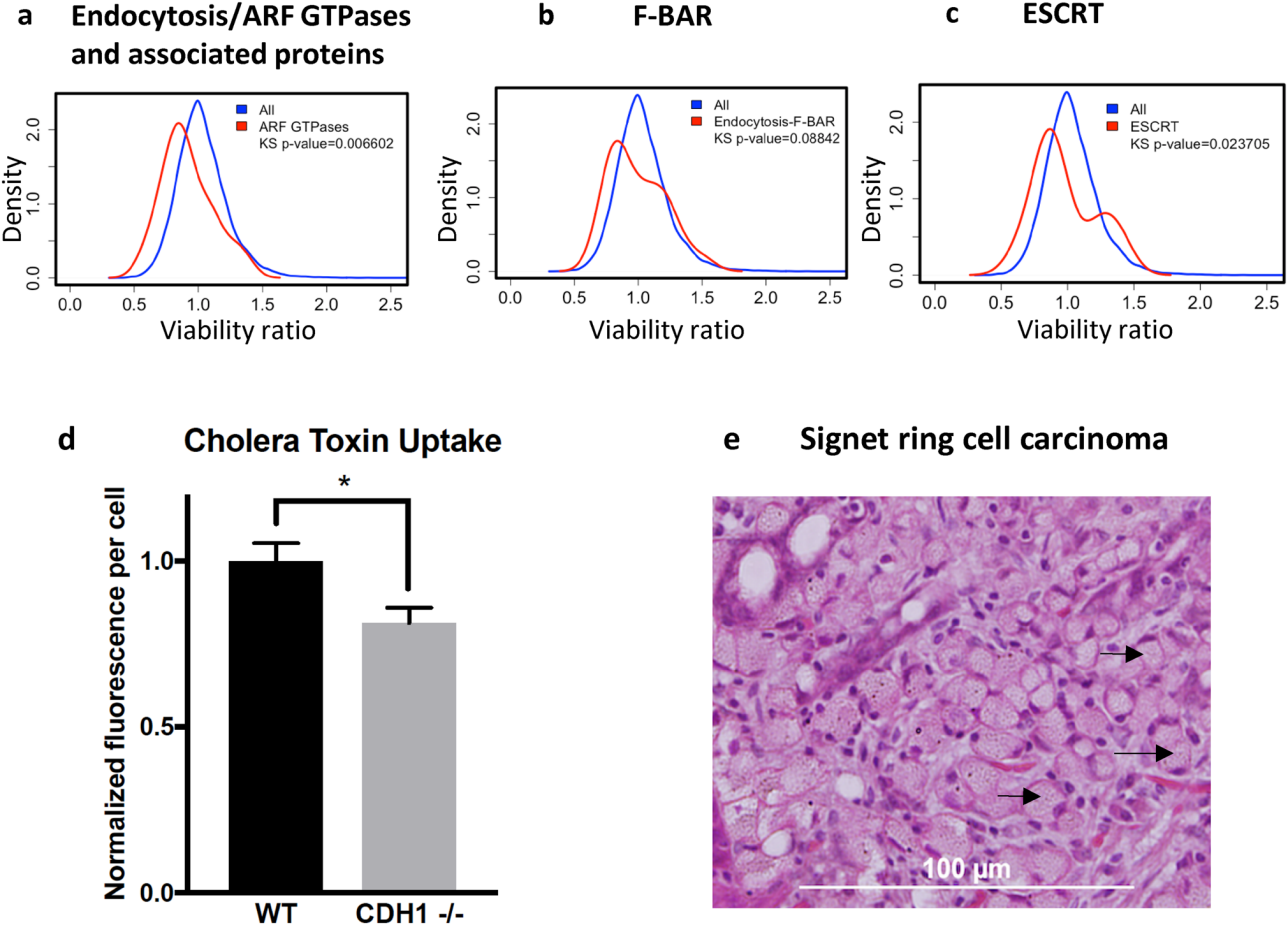
Density distributions of *CDH1*^−/−^/MCF10A viability ratios for **a** endocytosis/ARF GTPases and associated proteins, **b** F-BAR curvature proteins and **c** ESCRT curvature proteins. **d** Differential uptake of fluorescently labelled cholera toxin subunit B in MCF10A and *CDH1*^−/−^ cells. Cells were grown in medium deprived of cholera toxin (a standard component of MCF10A growth media) for 48 h before addition of Alexa Fluor 488-labelled cholera toxin B. After 30 min, cells were washed and fixed before cell counting and measurement of fluorescence intensity. Fluorescence was normalised to both the total cell count and the MCF10A result. **e** Haematoxylin and eosin stain of gastric stage T1a carcinoma from a germline *CDH1* mutation carrier showing mucin-filled signet ring cells. Three examples are indicated with black arrows

The highly significant SL and RSL effects of diverse GPCR’s, voltage-gated ion channels, and ubiquitinylation enzymes with roles in vesicle trafficking and receptor recycling, prompted us to investigate the possibility that disruption of plasma membrane organisation or dynamics in E-cadherin-null cells might unify these distinct effects. To do this, we first examined the density distributions of groups of proteins involved in membrane trafficking, using the lists provided in the KEGG Membrane Trafficking hierarchies [23]. 36 functional groups (with a minimum of 20 proteins/group) from the exocytosis, SNARE, endocytosis, endocytosis-lysozyme transport, protein recycling and endosome-golgi transport hierarchies were examined. The three most SL distributions, (i.e., the distributions with the lowest MCF10A*-CDH1*^−/−^/MCF10A density distribution peaks) comprised the family of ‘endocytosis/ARF GTPases and associated proteins’ (*n* = 23; peak = 0.85; *p* = 0.007; Fig. 3a) and two membrane curvature protein families: F-BAR (*n* = 20; peak = 0.84; *p* = 0.09; Fig. 3b) and ESCRT (*n* = 24, peak = 0.87; *p* = 0.02; Fig. 3c). These associations prompted us to test directly for the presence of an SL interaction between E-cadherin and endocytosis, by measuring the uptake of fluorescently labelled cholera toxin B in the MCF10A isogenic cell line pair. The B subunit of cholera toxin, which is non-toxic, is frequently used to measure internalisation by various endocytic mechanisms [24]. After 30 min, uptake of Alexa Fluor 488-labelled cholera toxin B was significantly lower (*p* = 0.04) in the *CDH1*^−/−^ cells compared to the wild-type MCF10A cells (Fig. 3d). Together, these data demonstrate that membrane trafficking is perturbed in MCF10A*-CDH1*^−/−^ cells, perhaps due to an underlying disruption of plasma membrane organisation and dynamics. Comparable disruption of exocytosis may explain the accumulation of mucins in signet ring cells, a common feature of DGC and a hallmark of early stage HDGC (Fig. 3e).

### Drug inhibition of membrane/cytoskeletal functions in breast and gastric isogenic cell lines

**Fig. 4:**
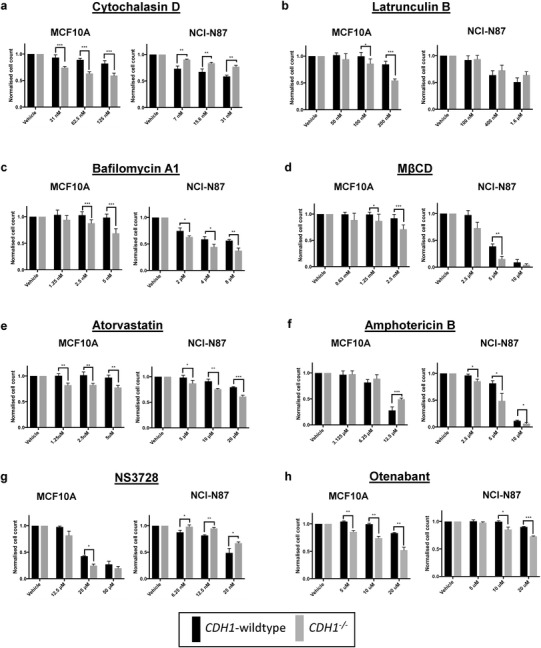
Normalised cell counts 48 h after treatment with serial dilutions of **a** cytochalasin D, **b** latrunculin B, **c** bafilomycin A1, **d** methyl β cyclodextrin, **e** atorvastatin, **f** amphotericin B, **g** NS3728 and **h** otenabant. For each drug, both the MCF10A and NCI-N87 isogenic pairs are shown (wild-type black bars; *CDH1*^−/−^ grey bars). Six fields/well at 4× magnification were captured using the Cytation 5 imager (Biotek). Nuclei were counted using Gen5 (Biotek) and normalised to the vehicle control for each cell line

To determine whether the disrupted cytoskeletal and membrane function of *CDH1*-deficient cells was reflected in altered drug sensitivity, we treated the isogenic MCF10A and gastric NCI-N87 cell lines with selected compounds that impact on cytoskeletal function, plasma membrane dynamics and lipid raft composition. The inhibitors of actin polymerisation, cytochalasin D and latrunculin B caused a significant SL differential (i.e., greater inhibition of the *CDH1*-deficient line) in the isogenic MCF10A cells but an RSL differential (i.e., greater inhibition of the *CDH1*-expressing line) was observed in the isogenic NCI-N87 cells (Fig. 4a, b). The endocytosis inhibitor bafilomycin A1, an antagonist of vacuolar ATPase [25], caused an SL differential in both the isogenic MCF10A and NCI-N87 lines (Fig. 4c). Lipid rafts are dynamic, relatively ordered membrane nanodomains that are enriched in cholesterol and sphingolipids and have a probable role in signal transduction [26]. Disruption of these domains with the membrane cholesterol-depleting drug methyl-β-cyclodextrin (MβCD) and the inhibitor of cholesterol synthesis, atorvastatin, caused an SL differential in both the MCF10A and NCI-N87 isogenic lines (Fig. 4d, e). Amphotericin B, an antifungal agent that binds to cholesterol and leads to perforation of the plasma membrane [27] showed contrasting effects, with an RSL effect in the MCF10A pair and an SL effect on the NCI-N87 isogenic lines (Fig. 4f). Regardless of the direction of the effect (SL or RSL), the differential activity of membrane-disrupting compounds strongly suggest that E-cadherin loss is leading to changes in plasma membrane dynamics. A fundamental shift in plasma membrane organisation or dynamics is consistent with our observation that RNAi inhibition has either an SL or RSL effect on the majority of GPCR and ion channel proteins. To explore the possible utility of pharmacological inhibition of these classes of membrane proteins, we tested the sensitivity of our isogenic cell line pairs to the chloride channel inhibitor NS3728 and the CB1 cannabinoid receptor antagonist Otenabant. NS3728 was SL in the MCF10A lines but RSL in the NCI-N87 lines, whereas Otenabant showed a significant SL effect on both isogenic pairs (Fig. 4g, h). These results point to the potential utility of selected GPCR or ion channel inhibitors for the treatment of *CDH1*-deficient cancers but highlight the importance of cellular context.

### Candidate synthetic lethal pathways identified using gastric tumour pathway metagenes

**Fig. 5:**
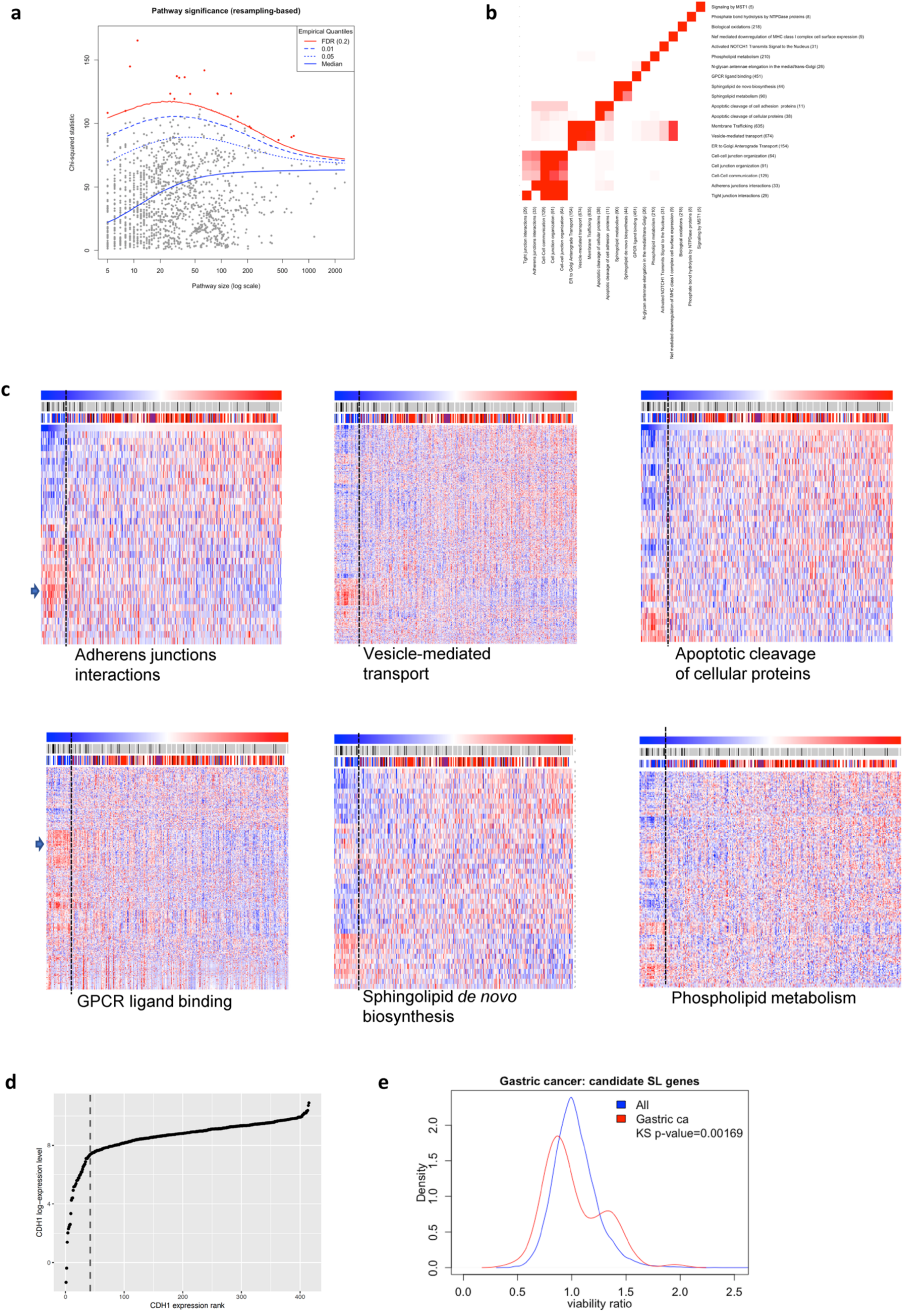
**a** Per-pathway chi-squared statistics for association between metagene and *CDH1* expression tertiles. Blue lines indicate quantiles of the test statistic distribution under the null hypothesis, derived via resampling (500,000 iterations). Solid red line indicates significance threshold relating to control of the False Discovery Rate (FDR) at 0.2. Red dots represent pathways with FDR-adjust *p* values below this threshold. **b** Overlap in gene content between each candidate SL pathway. **c** Hierarchical clustering of gene expression patterns from each of six candidate SL pathways. Samples (columns) are ordered from left to right based on *CDH1* expression (low to high) and the individual genes are in the rows. The Lauren classification of each tumour is marked in the bar immediately above the heatmap: blue-diffuse; red-intestinal; black-mixed; clear-undetermined. The middle bar shows *CDH1* mutation status for each sample: black, mutation (of any type); grey, no mutation; clear, missing data. The vertical dashed line shows the 10% rank position for tumour sample *CDH1* expression. The blue arrows mark two examples of clusters of genes with high relative expression in *CDH1*-deficient tumours. **d** Relative *CDH1* expression (log2) across the TCGA stomach cancer dataset. The 10% rank position is shown by the dashed line. Note, this line marks an inflexion point in the absolute E-cadherin expression level. **e** Density distributions of *CDH1*^−/−^/MCF10A viability ratios for 92 receptors and enzymes (excluding orphan GPCRs) that were present in the gene clusters with the highest relative expression in *CDH1*-deficient tumours

The isogenic gastric cancer and non-malignant breast cell line data provide valuable insights into possible targets for the chemoprevention and treatment of *CDH1*-deficient tumours. To enable us to prioritise functions and pathways that are most likely to be robust clinical targets, we statistically interrogated tumour gene expression data for patterns that were consistent with E-cadherin-associated synthetic lethality. In this approach, we hypothesised that if a genuine SL interaction exists between E-cadherin and any given pathway or protein, there would be fewer tumours than expected by chance with low expression (relative to all tumours) of both *CDH1* and the pathway or protein. RNA-seq data from 415 gastric cancers was obtained from TCGA [28] and expression metagenes were generated for each of the 2069 pathways in Reactome [29] (version 58) by taking the first eigenvector of the singular value decomposition [30]. Pathway metagene values were separated into tertiles, and a chi-squared test statistic was used to measure association with *CDH1* expression tertiles. Empirical *p* values were calculated for each pathway via resampling (500,000 iterations per pathway), and statistical significance was assessed after FDR correction (Fig. 5a). Using an adjusted *p* value threshold of 0.2, 20 pathways were identified as being associated with *CDH1* expression (Table 2), although several of these pathways overlapped in terms of gene composition (Fig. 5b). The majority of these candidate SL pathways were involved in cell–cell adhesion, membrane trafficking, membrane lipid composition, apoptosis of cell adhesion proteins and GPCR signalling. Consistent with the metagene selection method, hierarchical clustering of gene expression data from each of these pathways showed variably sized clusters of genes with either strikingly high or low expression in *CDH1*-deficient tumours (Fig. 5c). The expression changes were most pronounced in the 10% of tumours with the lowest *CDH1* expression by rank. Notably, the 10% rank level corresponded to a distinct inflexion point in the absolute *CDH1* expression of all samples (Fig. 5d). The 10% rank position corresponds to 36% of the overall median expression of *CDH1* and may mark a functional threshold for E-cadherin expression [31]. The low expression of *CDH1* in this bottom 10% of tumours cannot simply be attributed to high stromal cell contamination of DGCs, since tumours included in the TCGA analysis were restricted to those with a minimum tumour nuclei content of 60% [28]. The genes in the hierarchical clustering regions with the highest relative expression in *CDH1*-deficient tumours contained 92 known receptors or enzymes (excluding orphan GPCRs). The MCF10A/MCF10A *CDH1*^−/−^ RNAi cell viability ratios for these 92 genes showed a bimodal distribution, with the major SL peak occurring at a viability ratio of 0.87 (*p* = 0.002; Fig. 5e). This distribution demonstrates that our two approaches to identify candidate SL proteins produce an overlapping set of SL candidates which can now be prioritised for further validation (Suppl. Table 1).

**Table 2:** Gastric cancer pathways significantly associated with E-cadherin synthetic lethality

Reactome ID	Reactome pathway	Gene number^a^	FDR-adjusted *P* value
R-HSA-421270	Cell–cell junction organisation	64	0.0168
R-HSA-351906	Apoptotic cleavage of cell adhesion proteins	11	0.0168
R-HSA-1500931	Cell–cell communication	129	0.0228
R-HSA-164940	Nef-mediated downregulation of MHC class I complex cell surface expression	9	0.0228
R-HSA-5653656	Vesicle-mediated transport	674	0.0228
R-HSA-199991	Membrane trafficking	635	0.0228
R-HSA-428157	Sphingolipid metabolism	90	0.0315
R-HSA-446728	Cell junction organisation	91	0.0315
R-HSA-418990	Adherens junctions interactions	33	0.0315
R-HSA-2122948	Activated NOTCH1 Transmits Signal to the Nucleus	31	0.0315
R-HSA-111465	Apoptotic cleavage of cellular proteins	38	0.0315
R-HSA-1660661	Sphingolipid de novo biosynthesis	44	0.1146
R-HSA-199977	ER to Golgi Anterograde Transport	154	0.1442
R-HSA-500792	GPCR ligand binding	451	0.1453
R-HSA-975576	N-glycan antennae elongation in the medial/trans-Golgi	26	0.1453
R-HSA-8852405	Signalling by MST1	5	0.1669
R-HSA-420029	Tight junction interactions	29	0.1842
R-HSA-8850843	Phosphate bond hydrolysis by NTPDase proteins	8	0.197
R-HSA-211859	Biological oxidations	218	0.197
R-HSA-1483257	Phospholipid metabolism	210	0.197

^a^The number of genes in the pathway that were represented in the TCGA data

## Discussion

E-cadherin loss leads to distinct changes in plasma membrane organisation and cytoskeletal architecture. These changes are likely to be caused by disturbance of the close interaction between the membrane and the cortical actin network subsequent to E-cadherin loss [10, 32]. Actin interactions are required for membrane deformation processes such as endocytosis, exocytosis, autophagy and receptor/channel recycling [33–35]. They are also important for the maintenance of membrane characteristics such as membrane tension, ion channel activity and the correct partitioning of the membrane into lipid rafts or other nanostructures [32, 36–38]. We hypothesise that disorganisation of the cortical cytoskeleton in E-cadherin-deficient cells undermines the efficiency of these different processes, establishing numerous vulnerabilities that can be exposed with specific RNAi and chemical antagonists. We propose a model of E-cadherin synthetic lethality in which the function of both SL and RSL proteins is influenced by the presence of E-cadherin. For SL proteins, the loss of E-cadherin reduces functionality leading to an increased sensitivity to further inhibition of the SL protein (with either drug or RNAi). RSL proteins also experience reduced functionality in E-cadherin-deficient cells, but this loss in activity is compensated for by related proteins. As a result, further inhibition of an RSL protein has less effect on the viability of E-cadherin-deficient cells compared to E-cadherin-expressing cells. Predictions from this model include (i) non-specific antagonists of an RSL protein may inhibit related proteins and cause an SL effect; (ii) different cell types will differ in their repertoires of proteins with overlapping functions, resulting in the potential for contrasting SL or RSL effects with the same inhibitor; (iii) protein classes that are relatively promiscuous are more likely to be RSL (e.g. phosphatases vs kinases [16] and ubiquitin-specific peptidases vs RING E3 ubiquitin ligases), and (iv) protein complexes with functionally redundant subunits are likely to be RSL, consistent with our observations on spliceosomes, ribosomes and proteasomes. This model may not be exclusive. For example, the striking bimodality in the *CDH1*^−/−^/MCF10A viability ratios for the non-sensory GPCRs could be caused by biased agonism, a process whereby the same receptor can activate different downstream pathways depending on the ligand or allosteric transducers or modifiers proximal to the receptor [39, 40]. For GPCRs, the bias is typically between canonical G-protein signalling and signalling through a β-arrestin-mediated pathway [41]. Several allosteric modifiers have been linked to GPCR bias, including plasma membrane lipid composition [42, 43], membrane compartmentalisation [44], mechanical stretch [45] and the stoichiometry of downstream signalling components [46]. In conclusion, our insights into the mechanisms underpinning E-cadherin synthetic lethality obtained in both *in vitro* models and whole gastric tumours provide a framework for the rational selection of drugs that could be used to selectively target E-cadherin-deficient cancers. In particular, the disruption of PI3K/AKT signalling, membrane organisation and vesicle trafficking, points to important areas for future research.

## Electronic supplementary material

Below is the link to the electronic supplementary material.

Supplementary material 1 (XLSX 13 KB)

Supplementary material 2 (PPTX 588 KB)
