# Supplementary material for: E-cadherin-deficient cells have synthetic lethal vulnerabilities in plasma membrane organisation, dynamics and function
Source: Gastric Cancer. 2018 Jul 31;22(2):273–86. doi: 10.1007/s10120-018-0859-1 (PMC6394693; doi:10.1007/s10120-018-0859-1)
Supplement: Supplementary file 2 — Supplementary material 2 (PPTX 588 KB) [file 10120_2018_859_MOESM2_ESM.pptx]

## Slide 1
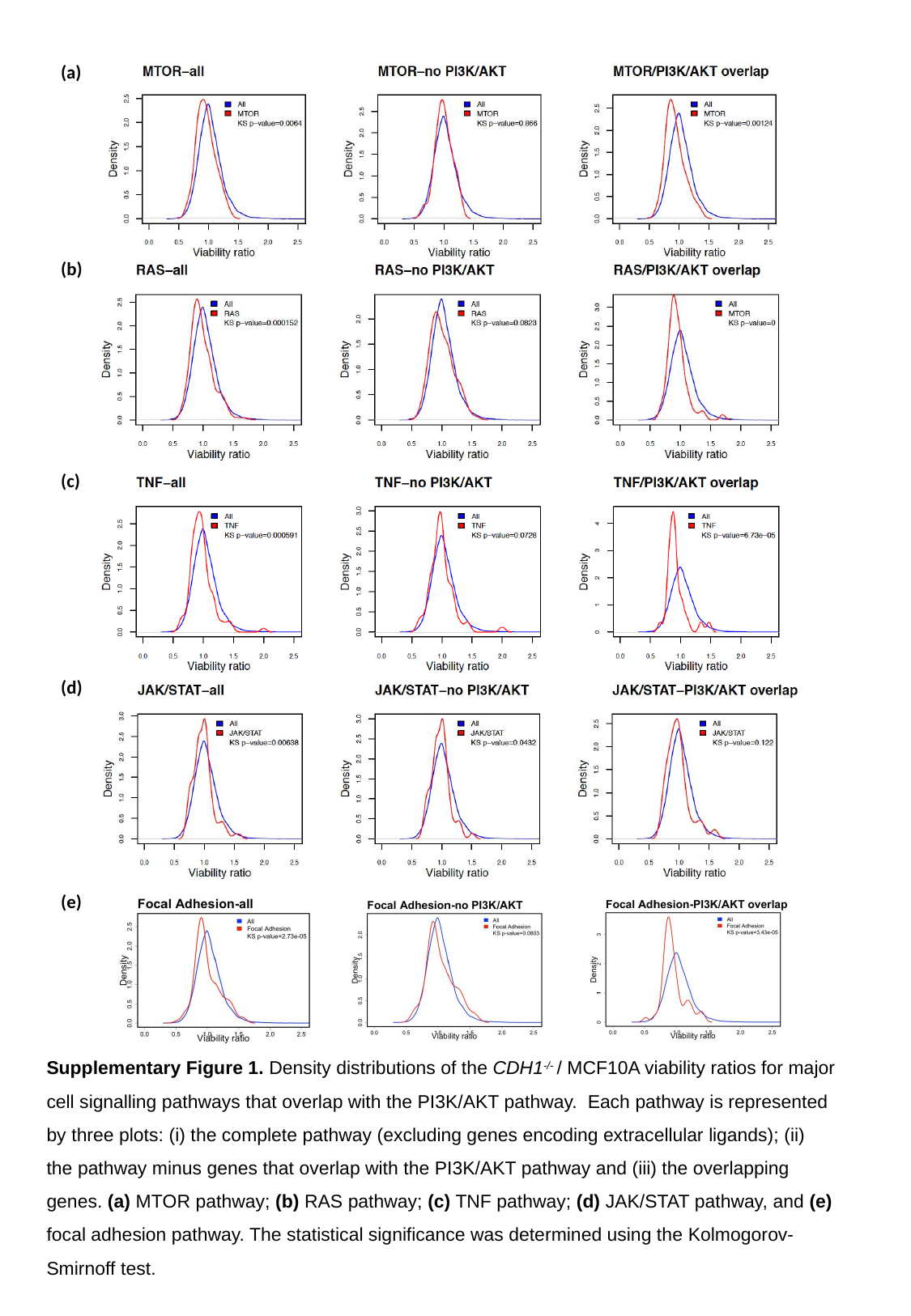

(a)
(b)
(c)
(d)
(e)
Supplementary Figure 1. Density distributions of the CDH1-/- / MCF10A viability ratios for major cell signalling pathways that overlap with the PI3K/AKT pathway. Each pathway is represented by three plots: (i) the complete pathway (excluding genes encoding extracellular ligands); (ii) the pathway minus genes that overlap with the PI3K/AKT pathway and (iii) the overlapping genes. (a) MTOR pathway; (b) RAS pathway; (c) TNF pathway; (d) JAK/STAT pathway, and (e) focal adhesion pathway. The statistical significance was determined using the Kolmogorov-Smirnoff test.
